# Formulation Optimization of Propranolol Hydrochloride Microcapsules Employing Central Composite Design

**DOI:** 10.4103/0250-474X.43024

**Published:** 2008

**Authors:** H. N. Shivakumar, R. Patel, B. G. Desai

**Affiliations:** Department of Pharmaceutical Technology, K. L. E. S’s College of Pharmacy, Rajajinagar, 2^nd^ Block, Bangalore-560 010, India

**Keywords:** o/o emulsion, propranolol hydrochloride, cellulose acetate butyrate, response surface methodology, central composite design

## Abstract

A central composite design was employed to produce microcapsules of propranolol hydrochloride by o/o emulsion solvent evaporation technique using a mixture of cellulose acetate butyrate as coat material and span-80 as an emulsifier. The effect of formulation variables namely levels of cellulose acetate butyrate (X_1_) and percentage of Span-80 (X_2_) on encapsulation efficiency (Y_1_), drug release at the end of 1.5 h (Y_2_), 4 h (Y_3_), 8 h (Y_4_), 14 h (Y_5_), and 24 h (Y_6_) were evaluated using the F test. Mathematical models containing only the significant terms were generated for each response parameter using multiple linear regression analysis and analysis of variance. Both the formulation variables exerted a significant influence (P <0.05) on Y_1_ whereas the cellulose acetate butyrate level emerged as the lone factor which significantly influenced the other response parameters. Numerical optimization using desirability approach was employed to develop an optimized formulation by setting constraints on the dependent and independent variables. The experimental values of Y_1_, Y_2_, Y_3_, Y_4_, Y_5_, and Y_6_ for the optimized formulation was found to be 92.86±1.56% w/w, 29.58±1.22%, 48.56±2.56%, 60.85±2.35%, 76.23±3.16% and 95.12±2.41%, respectively which were in close agreement with those predicted by the mathematical models. The drug release from microcapsules followed first order kinetics and was characterized by Higuchi diffusion model. The optimized microcapsule formulation developed was found to comply with the USP drug release test-1 for extended release propranolol hydrochloride capsules.

Sustained release Multi-particulate systems offer many advantages over single unit modified release systems. Some of the commonly reported advantages of multi-particulate systems include suitability for drug combinations, suitability for incompatible drugs, release of drug at different rate, reproducibility of gastric emptying^1^, better statistical assurance of drug release and less likelihood of dose dumping^2^, less inter and intra patient variability, less variation in gastric transit time^3^ and gastric emptying that is relatively independent of the nutritional status^4^. Microencapsulation techniques have been one of the many approaches employed to produce peroral polymeric multiparticulate systems^5^. Most of these techniques have used lipophilic drugs, since hydrophilic drugs usually exhibit low loading efficiency^6^. Considering this, several methods like phase separation^7^, spray drying^8^, and solvent evaporation^9^ procedures have been proposed to entrap water-soluble drugs. However, the problem with phase separation technique is the aggregation and residual solvent in the resulting microspheres whereas, spray drying technique exposes the drug to higher temperatures. The conventional oil in water solvent evaporation techniques cannot be used for water-soluble drugs as they result in low loading efficiency due to partitioning of the drug into the continuous phase^9^. To reduce the drug partitioning into the continuous phase and to enhance the drug loading o/o solvent evaporation methods have been proposed of late^10^. Propranolol hydrochloride, a nonselective β-adrenergic blocking agent, is widely used in the treatment of hypertension, angina pectoris and other cardiovascular disorders^11^. It is almost completely absorbed following oral administration but its bioavailability has been limited due to extensive first pass metabolism. The short biological half-life (3-6 h) and high frequency of administration (40 to 80 mg 2 to 3 times a day) initiated the need to develop once a day controlled release formulation.

The current investigation was undertaken to systematically analyze the effect of different formulation variables on the microcapsule properties using a response surface methodology and finally develop an optimized formulation with desirable features. Cellulose acetate butyrate, a commonly employed as a coat material in microencapsulation was used as a rate controlling polymer whereas span-80 was used as an emulsifier to stabilize the o/o emulsion produced.

Propranolol hydrochloride was generous sample from Zydus Health Care Ltd., Bangalore, India. Cellulose acetate butyrate (CAB; MW: 30,000) was purchased from Rolex chemical industries, Mumbai, Span-80, potassium dihydrogenorthophosphate, disodium hydrogenorthophosphate and sodium hydroxide were purchased from S. D. Fine Chemicals, Mumbai. All other chemicals and regents used were of analytical grade.

A rotatable central composite design was employed to produce controlled release microcapsules of propranolol hydrochloride by o/o emulsion solvent evaporation technique^12^. The design consisted of four full factorial points (F1 to F4), four axial points (A1 to A4) and five center points (C1 to C5). The initial polymer loads in the microcapsules (X_1_) and the concentration of span used during emulsification (X_2_) were the two independent variables analyzed. The dependent variables investigated were percent encapsulation efficiency (Y_1_), and the drug release at 1.5h (Y_2_), 4h (Y_3_), 8h (Y_4_), 14h (Y_5_) and 24h (Y_6_). The selected factor combinations indicating the actual and coded levels as per the design are represented in Table 1.

**TABLE 1:** FACTOR COMBINATIONS FOR THE MODEL MICROCAPSULE FORMULATIONS PREPARED ACCORDING TO ROTATABLE CENTRAL COMPOSITE DESIGN

Batch	Factor levels	
	
	X_1_	X_2_
F1	-1(40)	-1(0.50)
F2	1(70)	-1(0.50)
F3	-1(40)	1(1.50)
F4	1(70)	1(1.50)
A1	-1.41(33.79)	0(1.00)
A2	+1.41(76.21)	0(1.00)
A3	0(55)	-1.41(0.29)
A4	0(55)	+1.41(1.71)
C1	0(55)	0(1.00)
C2	0(55)	0(1.00)
C3	0(55)	0(1.00)
C4	0(55)	0(1.00)
C5	0(55)	0(1.00)

Factor X_1_ represents initial polymer loads (%w/w) and Factor X_2_ represents span-80 concentrations (%w/w). The parentheses represent the decoded values of the factors.

Drug loaded CAB microcapsules were produced by o/o emulsion solvent evaporation technique^13^. Liquid paraffin (70 g) was mixed with span-80 in a beaker for 15 min to obtain a homogenous oily phase. An accurately weighed amount of propranolol hydrochloride screened was dispersed for 10 minutes in solution of CAB in acetone using a cyclomixer (CM 101, Remi Equipments Ltd. Mumbai). The resulting dispersion (20 ml) was slowly added to oily phase and stirred at a speed of 1200 rpm for 15 min using a propeller stirrer (RQ 121 D, Remi Equipments Ltd., Mumbai). The stirring was continued at 900 rpm till the acetone was completely evaporated to produce the drug-loaded microcapsules. The microcapsules produced were filtered, washed with petroleum ether to remove the traces of liquid paraffin and dried at the room temperature for 24 h. A total of 13 batches of microcapsules were produced by varying the initial polymer loads and the concentration of Span-80 used during emulsification. The processing variable like the stirring speed and other formulation variables such as amount of liquid paraffin, and the volume of the CAB solution incorporated were maintained constant for all the batches produced.

The projected diameter of a total of 200 microcapsules from each batch was determined by optical microscopy. The geometric mean diameter (d_g_) and standard deviation (σ_g_) were computed by fitting the number distribution data into log normal plots.

An accurately weighed sample of the microcapsules from each batch were triturated in a mortar, transferred to a standard volumetric flask and sonicated with 0.1 N HCl for 15 min (Enertech Electronics Pvt. Ltd., Mumbai) to extract the drug. The resulting dispersions were filtered, suitably diluted and assayed at 290 nm in a Shimadzu 1700 UV/Vis spectrophotometer (Shimadzu Corporation, Kyoto, Japan). The percent drug content and percent microencapsulation efficiency of different batches of microcapsules was calculated from the assay values using a standard curve prepared in the same solvent.

The *in vitro* dissolution studies of the microcapsules were performed for a period of 24 h in USP XXIII dissolution rate test apparatus-1 (TDT-08 T, Electrolab (I) Ltd., Mumbai, India) following USP drug release test-1^14^. The studies were carried out initially in 900 ml of 0.1 N HCl (pH 1.2) for the first 1 hours followed by phosphate buffer of pH 6.8 for rest of the period. Microcapsules containing 100 mg of the drug were loaded in the basket of the apparatus and rotated at stirring speed of 100 rpm in the dissolution media maintained at 37±0.5°. Aliquots of samples were withdrawn at 1.5, 4, 8, 14 and 24 h, passed through 0.45-μm filter, diluted suitably, and assayed spectrophotometrically at 290 nm. The raw dissolution data recorded in triplicate was analyzed to calculate the amount of drug released and percentage cumulative drug released at different time intervals. The microcapsules comply with the USP drug release test-1 for extended release propranolol hydrochloride capsules only if the drug release at the end of 1.5, 4, 8, 14 and 24 h ranged from 15 to 30, 30 to 60, 55 to 80, 70 to 95 and 80 to 110%, respectively.

The targeted response parameters were statistically analyzed by applying one-way ANOVA at 0.05 level in Design-Expert® software (version 6.0.5 Stat-Ease Inc., Minneapolis, MN, USA)^15^. The individual parameters were evaluated using the Fischer test and quadratic models of the form

$$Y=\beta_{0}+\beta_{1}X_{1}+\beta_{2}X_{2}+\beta_{3}X_{1}X_{2}+\beta_{4}{X_{1}}^{2}+\beta_{5}{X_{2}}^{2}$$

were generated for each response parameter using multiple linear regression analysis (MLRA) and analysis of variance (ANOVA). Y stands for the level of the measured response; β_0_ is the intercept; β_1_ to β_5_ are the regression coefficients. X_1_ and X_2_ stand for the main effects; X_1_ X_2_ is the interaction between the main effects; X_1_^2^ and X_2_^2^ are the quadratic terms of the independent variables that were used to simulate the curvature of the designed sample space. Predictor equations containing only the significant terms were generated using a backward elimination procedure. A numerical optimization procedure using desirability approach was used to locate the optimal settings of the formulation variables in view to obtain the desired response^15^. Constraint for the drug release at 1.5, 4, 8, 14 and 24 h were set in the range of 0-30, 30-60, 55-80, 70-95 and 80-110%, respectively as per the USP drug release test-1. Maximizing the microencapsulation efficiency was also set as a goal to locate the optimum settings of the independent variables in the new formulation. The optimized formulation was evaluated for the response parameters and the experimental values obtained were compared with those predicted by the mathematical models.

SEM photomicrographs under lower magnification (40 X) indicated that the microcapsules were discrete, spherical and uniform in shape (fig. 1a). Higher magnifications (75 X) revealed that the microcapsules had a rough surface without any evidence of surface drug (fig. 1b) suggesting efficient encapsulation of the drug in the CAB polymeric matrix. This may be due to the fact that the drug is thoroughly wetted, finely dispersed and enveloped within the polymer matrix prior to encapsulation.

**Fig. 1:**
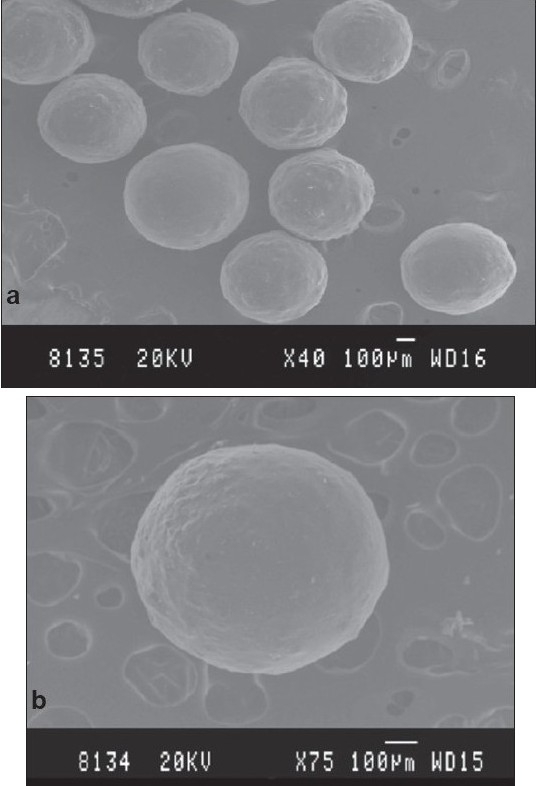
SEM Photomicrographs Scanning photomicrographs of propranolol hydrochloride microcapsules under (a) low magnification and (b) high magnification.

The number distribution data of different batches of microcapsules obtained from optical microscopy indicated a log normal distribution with the geometric mean diameters ranging between 398.10 μm and 912.01 μm. The microcapsule size was found to increase with increased polymer loads and decrease with increases Span-80 concentrations. The increase in the microcapsule size with increase in the polymer levels can be attributed to the increase in the viscosity of the polymer solution as a consequence of increased polymer concentrations at higher polymer levels. Span-80 by its emulsifying actions was found to reduce the emulsion droplet size during emulsification and thereby the microcapsule size.

The microencapsulation efficiency was found to depend on the initial polymer loads and the concentration of Span-80 employed during emulsification. The high values of the encapsulation efficiencies (Table 2) justified the use of o/o emulsification technique for encapsulation of the model water-soluble drug. The linear model generated for encapsulation efficiency (Table 3) was significant and indicated that both the main factors exerted a significant positive influence on the encapsulation efficiency. The increase in microencapsulation efficiency with the increase in polymer concentrations could be due to better encapsulation of the drug at high polymer loads and decrease in the amount of surface drug. The increase in the drug encapsulation with increase in the span-80 levels could also be ascribed to the emulsifying effect of the surfactant which helped the drug to be finely dispersed and embedded in the polymer matrix prior to encapsulation. In addition to the main factors the two-way interaction had a negative influence on the encapsulation efficiency.

**TABLE 2:** RESPONSE PARAMETERS OF MODEL MICROCAPSULE FORMULATIONS PREPARED ACCORDING TO ROTATABLE CENTRAL COMPOSITE DESIGN

Batch	Y_1_	Y_2_	Y_3_	Y_4_	Y_5_	Y_6_
F1	82.50±1.23	72.66±3.17	92.46±2.82	99.07±1.22	100.02±0.12	100.00±0.08
F2	90.45±0.92	23.86±1.52	45.80±2.48	56.80±2.33	72.84±3.62	94.66±2.23
F3	96.23±0.89	89.12±2.24	99.60±1.26	100.00±0.22	100.00±0.22	100.00±0.22
F4	94.90±1.42	24.32±1.36	44.82±2.95	55.98±1.86	75.61±1.46	95.96±2.16
A1	86.55±0.93	86.76±2.32	98.07±1.84	100.06±0.45	100.02±0.23	100.02±0.15
A2	96.70±1.53	20.92±1.78	43.60±2.04	56.38±2.21	74.42±3.25	92.32±3.15
A3	86.75±0.85	55.62±2.16	89.20±2.84	100.02±0.23	100.02±0.23	100.02±0.23
A4	95.42±1.34	64.71±3.17	90.76±2.09	98.59±1.67	100.00±0.75	100.00±0.75
C1	91.55±1.32	54.12±1.85	80.14±1.87	96.41±2.34	100.00±0.86	100.00±0.86
C2	90.95±1.05	53.65±1.82	79.15±2.93	93.58±2.85	100.00±0.76	100.00±0.76
C3	90.20±0.78	54.76±2.36	79.13±2.79	92.98±1.12	100.00±0.86	100.00±0.86
C4	88.75±0.82	52.98±2.75	78.10±2.28	85.48±3.45	100.00±0.65	100.00±0.65
C5	90.12±1.52	48.64±2.16	74.07±2.69	87.88±2.49	100.00±0.84	100.00±0.84

Y_1_ stands for percentage encapsulation efficiency; Y_2_, Y_3_, Y_4_, Y_5_ and Y_6_ represent the percentage drug released at the end of 1.5 h, 4.0 h, 8.0 h, 14.0 h and 24 h of dissolution. All the values represent mean ± standard deviation of three determinations.

**TABLE 3:** MATHEMATICAL MODELS GENERATED FOR DIFFERENT RESPONSE PARAMETERS

SI. No.	Model	F-value	p > F	R_2_
1.	Y_1_ = 90.85+2.62X_1_ + 3.81X_2_ - 2.32X_1_X_2_	30.14	<0.0001	0.91
2.	Y_2_ = 55.16 - 23.35X_1_	149.58	<0.0001	0.93
3.	Y_3_ = 77.30 - 22.31 X_1_	53.69	<0.0001	0.91
4.	Y_4_ = 91.48 - 18.00 X_1_ - 8.60X_1_^2^	41.87	<0.0001	0.90
5.	Y_5_ = 98.96 - 10.72 X_1_ - 7.70 X_1_^2^	51.22	<0.0001	0.91
6.	Y_6_ = 99.75 -1.82 X_1_ - 1.23 X_1_^2^	256.16	<0.0001	0.98

Y_1_ stands for percentage encapsulation efficiency; Y_2_, Y_3_, Y_4_, Y_5_ and Y_6_ represent the percentage drug released at the end of 1.5 h, 4.0 h, 8.0 h, 14.0 h and 24 h of dissolution. *The values represent the average of three determinations (n=3).

The drug released at different time intervals from model formulations are displayed in Table 2. The formulations representing the factorial points F1 and F3 were characterized by an initial rapid release phase followed by a slow release phase. The initial burst effect contributing to 72.66±3.17% and 89.12±2.24% of the release was observed from formulations F1 and F3 respectively in pH 1.2. The low polymer settings in these formulations resulted in more drug remaining at the surface of the microcapsules, which produced an initial rapid release phase. A burst release was not evident in formulations F2 and F4 that represented the other factorial points. These formulations were able to sustain the drug for a period of 24 h in a more progressive manner. This could be due to higher polymer setting, which resulted in better encapsulation of the drug within the microcapsules.

A initial rapid drug release in pH 1.2 (86.76±2.32%) was observed with the formulations representing axial point A1 which released most of the drug within 4 h of dissolution. This could be due to the lowest CAB setting (33.79% w/w), which leads to more drug being retained on the surface. The evidence of the burst release from the formulations A3 and A4 and their inability to sustain the drug release beyond 8 h of dissolution suggested that the intermediate polymer settings (55% w/w) might not sufficiently prevent the initial rapid drug release and prolong the drug release considerably. The formulations representing axial point A2 that had the highest polymer load (76.21%) was successful in controlling the drug release for the entire dissolution period of 24 h with out initiating a rapid release phase.

To determine the lack of fit from the generated mathematical model, five replicates of the formulations representing the center points (C1 to C5) were included in the design to determine the pure error due to the experimental procedures. Clustering and overlapping of the release profiles indicated that the errors caused by the experimental procedures were negligible in generating a meaningful data fitment for the response parameters. All the center points were characterized by an initial rapid release and were only successful in sustaining the drug release for a period of 8 h. Of the 13 design points, formulations F2, F4 and A2 were found to comply with the USP drug release test-1 for extended release propranolol hydrochloride capsules. Higher polymer loads (> 70% w/w) in these batches resulted in better encapsulation of the drug and decreased the amount of surface drug. This in turn was found to minimize the burst release and simultaneously prolong the drug release for 24 h.

The linear models generated for the release parameters Y_2_ Y_3_, Y_4_, Y_5_ and Y_6_ was found to be significant (Table 3) and indicated that polymer levels emerged as the lone factor, which significantly influenced these release parameters.

The mechanism of drug release from these three batches was characterized to be diffusion controlled as plots of percent cumulative drug release versus square root of time was found to be linear. The values of the Higuchi rate constants for the batches F2, F4 and A2 were found to be 18.01±0.52, 19.01±0.034 and 19.05±0.066 h^-1/2^ with the R^2^ values of 0.975±0.005, 0.988±0.006 and 0.977±0.004, respectively. The kinetics of drug release from microcapsules was found to follow first order kinetics as the plots of log concentration of drug retained versus time were linear. The values of the first order rate constants for the batches F2, F4 and A2 were found to be 0.010±0.01, 0.11±0.02 and 0.09±0.01 h^-1^ with the R^2^ values of 0.924±0.003, 0.918±0.004 and 0.971±0.006, respectively.

A numerical optimization technique using the desirability approach was employed to develop a new formulation with the desired responses. An optimized formulation was developed using 70% w/w of CAB and 30% w/w of propranolol hydrochloride using 1.00% w/w span-80 during emulsification. The optimized formulation was evaluated for microencapsulation efficiency and drug release at different time intervals. The optimized formulation developed was found to comply with the USP drug release test-1. Table 4 portrays the value of the observed responses and those predicted by mathematical models long with the percentage prediction errors. The low values of the prediction error for the response parameters establish the high forecasting ability of response surface methodology. The drug release from the optimized formulation was found to follow first order kinetics and was characterized by Higuchi diffusion model.

**TABLE 4:** COMPARISON OF THE EXPERIMENTALLY OBSERVED RESPONSES OF THE OPTIMIZED MICROCAPSULE FORMULATION WITH THE PREDICTED RESPONSES

Response parameters	Constraints set	Observed values*	Predicted values	% Error
Y_1_	Maximize	92.86 ± 2.15	93.47	0.66
Y_2_	< 30%	29.58 ± 1.57	29.47	0.37
Y_3_	30 to 60 %	48.56 ± 2.56	51.71	-6.48
Y_4_	55 to 80%	60.85 ± 2.42	64.28	-5.63
Y_5_	70 to 95%	76.23 ± 2.87	80.10	-4.88
Y_6_	80 to 110%	95.12 ± 2.74	95.38	-0.27

Y_1_ stands for percentage encapsulation efficiency; Y_2_, Y_3_, Y_4_, Y_5_ and Y_6_ represent the percentage drug released at the end of 1.5 h, 4.0 h, 8.0 h, 14.0 h and 24 h of dissolution. *The values represent the average of three determinations (n=3).

A o/o emulsion solvent evaporation technique has been successfully employed to produce cellulose acetate butyrate microcapsules of propranolol hydrochloride with high encapsulation efficiency and desirable release profiles. Both the formulation variables analyzed exerted a significant influence on the encapsulation efficiency whereas the polymer levels emerged as the lone factor, which influenced the drug release. The optimized microcapsules formulation developed employing desirability approach exhibited a drug release that complied with the USP drug release test-1 and also demonstrated high encapsulation efficiency. The results obtained indicate that response surface methodology can be successfully used to quantify the effect of several formulation variables and develop an optimized formulation thereby minimizing the number of experimental trials and cutting down the formulation development cost.

## References

[1] Amighi K, Timmermans KJ, Puigdevall Baltes JE, Moes AJ (1998). Peroral sustained-release film-coated pellets as a means to overcome physicochemical and biological drug related problems: Part 1, *In vitro* development and evaluation. Drug Develop Ind Pharm.

[2] Lordi NG, Lachmann L, Lieberman HA, Kanig JL (1986). Sustained release dosage forms. The theory and practice of Industrial Pharmacy.

[3] Follonier N, Doelker E (1992). Biopharmaceutical comparison of oral multi-unit and single-unit sustained release dosage forms. STP Pharm Sci.

[4] Davis SS (1986). Evaluation of the gastrointestinal transit of pharmaceutical dosage forms using the technique of gamma scintigraphy. STP Pharm Sci.

[5] Benita S (1996). Survey of microencapsulation processes. Microencapsulation: Methods and industrial applications.

[6] Lee JH, Park TG, Choi HK (2000). Effect of formulation and processing variables on the characteristics of microspheres for water soluble drugs prepared by w/o/o double emulsion solvent diffusion method. Int J Pharm.

[7] Lameiro MH, Lopez A, Martins LO, Alves Pm, Melo E (2006). Incorporation of model protein into chitoson bile salts microparticles. Int J Pharm.

[8] Cevhar E, Orphan Z, Malazimoglu L, Sensoy D, Alper M, Yildiz A (2006). Characterization of biodegradable microspheres containing vancomycin and treatment of experimental osteomyelitis caused by methicillin resistant *S. aureus* with prepared microspheres. Int J Pharm.

[9] Al-Maaieh A, Flanagan DR (2005). New drug salt formation in biodegradable microspheres. Int J Pharm.

[10] Lamprecht A, Yamamoto H, Tacheuchi H, Kawashima Y (2005). Observations in simultaneous microencapsulation of 5-fluorouracil and leucovorin for combined pH dependent release. Eur J Pharm Biopharm.

[11] Sweetman SC (2005). Martindale The complete drug reference.

[12] Lewis GA, Mathieu D, Phan Tan Lui R (1999). Pharmaceutical experimental design.

[13] Rodriguez M, Vila-Jato JL, Torres D (1998). Design of a new multiparticulate system for potential site-specific and controlled drug delivery to the colonic region. J Control Release.

[14] (2000). The United State Pharmacopoeia 24.

[15] Narendra C, Srinath MS, Prakash Rao B (2005). Development of three layer buccal compact containing metoprolol tartrate by statistical optimization technique. Int J Pharm.

